# Effects of food insecurity risk on infant feeding confidence and practices during the COVID-19 pandemic and its waves

**DOI:** 10.1186/s13006-026-00838-5

**Published:** 2026-04-11

**Authors:** Maha Hussain, Jessica Hyun, Elena Arduin, Margaret H. Kyle, Wanda Abreu, Tessa Scripps, Adrita Khan, Melissa Glassman, Melissa S. Stockwell, Lauren Walzer, Dani Dumitriu, Cristina R. Fernández

**Affiliations:** 1 Department of Pediatrics, Columbia University Vagelos College of Physicians and Surgeons, 622 W 168th St, VC4-417, New York, NY 10032 USA; 2 Department of Population and Family Health, Columbia University Mailman School of Public Health, New York, NY USA; 3 Department of Psychiatry, Columbia University Irving Medical Center, New York, NY USA; 4 NewYork-Presbyterian Hospital, New York, USA

**Keywords:** Food insecurity, Maternal confidence, Infant feeding, Pregnancy outcomes, SARS-CoV-2

## Abstract

**Background:**

The COVID-19 pandemic and its aftermath changed numerous social and economic environments. Food insecurity rates rose with potential negative ramifications for infant feeding methods. The relationship between food insecurity and infant feeding practices since the onset of the pandemic is not yet fully understood. We examined these relationships in a cohort of infants born during the onset and aftermath of the COVID-19 pandemic in New York City.

**Methods:**

We conducted a cross-sectional analysis of infants enrolled from birth into the COVID-19 Mother-Baby Outcomes (COMBO) study and born March 2020 to May 2024. We measured food insecurity risk in the prior 30 days with a 2-item survey adapted from Hunger Vital Sign™, infant feeding confidence at hospital discharge, and current infant feeding practices. Associations between food insecurity and infant feeding outcomes were examined using multivariable regression models.

**Results:**

In our sample, 40% of women had been exposed to prenatal SARS-CoV-2 infection and approximately 24% of mothers were experiencing food insecurity. Food insecurity risk was significantly associated with prenatal SARS-CoV-2 infection, Spanish as one’s preferred language, and self-identifying as Latina. On unadjusted models, food insecurity was associated with formula feeding, with 44% of mothers experiencing food insecurity opting to formula feed vs. 28% of food secure mothers (*p* < 0.001), but this relationship was no longer significant after adjusting for covariates (*p* = 0.06). On adjusted models stratified by early (March 2020-December 2021) and later waves of the pandemic (January 2022-May 2024), food insecurity was significantly associated with increased formula feeding.

**Conclusions:**

Food insecurity was associated with infant feeding practice on the unadjusted model of the entire sample but not after adjusting for confounders. Food insecurity risk was significantly associated with lower odds of exclusive breastfeeding in each distinct wave of the pandemic on stratified analyses. These findings suggest potential influence of unmeasured, fluctuating external factors across the 2020–2024 time period, such as health and social services policy changes, social support variations, and the 2022 formula shortage. Our findings suggest a continued need for targeted screening of postpartum mothers for food insecurity and targeted services that support exclusive breastfeeding, especially among food-insecure mothers.

**Supplementary Information:**

The online version contains supplementary material available at 10.1186/s13006-026-00838-5.

## Background

Breastfeeding is the optimal method of infant feeding, yet only 25.8% of women in the U.S. meet the recommendation to exclusively breastfeed for the first six months [1–5]. The World Health Organization advises exclusive breastfeeding for the first 4–6 months, followed by continued breastfeeding with complementary foods until age two [6]. Breastfeeding offers substantial health benefits, while lower rates are linked to poorer maternal and infant outcomes [7, 8].

The COVID-19 pandemic further reduced breastfeeding rates. A United Kingdom study found that 18.9% of mothers stopped breastfeeding due to lack of support, with minority mothers disproportionately affected [9]. Another study reported that women who gave birth during the pandemic were 15% less likely to exclusively breastfeed [10]. In our previous research from the COVID-19 Mother Baby Outcomes (COMBO) longitudinal study, we observed that mothers with prenatal SARS-CoV-2 infection were less likely to breastfeed in the early postpartum period, despite hospital policies encouraging rooming-in and direct breastfeeding [11]. While early national guidance discouraged direct contact, our institution promoted skin-to-skin care and breastfeeding with proper hygiene, due to limited evidence of vertical transmission [12, 13]. These findings suggest that broader social and economic factors related to the pandemic—not just infection status—may influence infant feeding practices.

Food insecurity is a global public health challenge that was exacerbated by the COVID-19 pandemic [14]. Food insecurity is defined as when nutritionally adequate and safe foods are unavailable or when the ability to acquire such foods in socially acceptable ways is limited or uncertain [15]. Though food insecurity has always been a challenge in the United States, rates of household food insecurity in the nation increased dramatically increased from 11% in 2019 to 38% in March 2020 [16, 17]. In April 2020, 35% of households with children under 18 reported experiencing food insecurity [17]. In 2022 and 2023, food insecurity rates were reported to be 12.8% and 13.5% respectively across the USA [18]. Though there has been a drop in food insecurity rates since the onset of the pandemic, the rate remains higher than pre-pandemic levels, posing significant concerns.

New York State’s March 2020 pandemic stay-at-home orders disrupted economic life, as record-high unemployment, supply shortages, and income insecurity produced both independent and interrelated effects. In addition to economic hardship, many postpartum mothers experienced social isolation and reduced mobility due to fears of COVID-19 exposure; although the first COVID-19 vaccines were eligible for use by the general public in April 2021, differences in vaccine uptake, mask-wearing, and employer requirements may have continued to challenge regular access to economic stability, food, healthcare, and breastfeeding support [19]. These interactions likely contributed to the significant rise in food insecurity observed during the pandemic, with many households experiencing food insecurity for the first time [20, 21]. While the economic and social disruptions caused by the COVID-19 pandemic are well-documented, it is unclear how infant feeding practices may have been influenced by food insecurity during this time. As a result of the COVID-19 pandemic, parents experienced heightened levels of stress, with mothers likely bearing a disproportionate share of the burden, which may have affected infant feeding practices [22–27]. Though some studies have shown an association between higher levels of household food insecurity and decreased breastfeeding rates prior to the COVID-19 pandemic [28–30], other studies did not find a significant association [31, 32], suggesting that this relationship may be influenced by the particular contexts in which the maternal-infant dyad live.

One potential mechanism linking food insecurity to infant feeding practices is maternal feeding confidence. Chezem et al. conceptualize maternal feeding confidence as a mother’s belief in her ability to successfully breastfeed and manage feeding-related challenges, noting that confidence is shaped by knowledge, experience, and support and is strongly associated with breastfeeding intentions and duration [33]. Experiencing food insecurity has been associated with lower levels of maternal self-efficacy [34], though additional research is needed to clarify how food insecurity influences maternal feeding confidence specifically and how this relationship may vary across different social and economic contexts.

The onset and aftermath of the COVID-19 pandemic is a novel context; though it appears that breastfeeding rates decreased during the early pandemic period, it is not yet well understood how the relationship between food insecurity and infant feeding may have evolved as the social environment changed. Each wave of the COVID-19 pandemic was characterized by different levels of economic instability, healthcare access, social support, and public health restrictions, all of which may differentially influence food insecurity and infant feeding decisions. Early waves were marked by acute disruptions and uncertainty, while later waves reflected more chronic stressors and prolonged resource strain. Accounting for these temporal differences allows interventions to be better timed and tailored to the specific needs families face at different stages of crisis. Further understanding of the associations between food insecurity in the context of the early and later waves of the COVID-19 pandemic and maternal-infant feeding practices is crucial to inform programs and interventions that seek to improve breastfeeding rates among families experiencing hardships.

The primary objectives of our study were to determine the association between risk for household food insecurity and breastfeeding practices and maternal feeding confidence during the COVID-19 pandemic and its aftermath in New York City (NYC). Secondary objectives of our study were to examine the association between food insecurity and breastfeeding practices between the early and later time periods of the COVID-19 pandemic. We hypothesized that mothers experienced food insecurity during the pandemic would be less likely to breastfeed and report lower infant feeding confidence.

## Methods

### Study design, study site, and study population

Our cross-sectional study was nested within Columbia University Irving Medical Center’s (CUIMC) COVID-19 Mother Baby Outcomes (COMBO) ongoing prospective cohort study, investigating the health and development of mother-infant dyads from onset of the COVID-19 pandemic in March 2020. History of maternal prenatal SARS-CoV-2 infection was determined by nasopharyngeal PCR and serological testing for SARS-CoV-2 antibodies. Mother-infant dyads with history of maternal SARS-CoV-2 infection (exposed) were matched to unexposed dyads with a comparable infant sex, gestational age at birth, mode of delivery and date of birth who tested negative for SARS-CoV-2 at the time of delivery and had no record of maternal SARS-CoV-2 infection before or during pregnancy. Dyads enrolled into COMBO at infants’ birth to 2 months of age. Participants were recruited by research assistants and clinicians via phone calls, SMS, and emails, and then were asked to complete 30-60-minute self-administered surveys. Eligibility criteria for the present study include COMBO-enrolled English- and/or Spanish-speaking mothers who gave birth from March 2020 to May 2024 at CUIMC/NewYork-Presbyterian Hospital (NYP).

CUIMC/NYP is located in the Washington Heights/Inwood community of northern Manhattan and handles 6,000–7,000 live births per year. 46% of residents of this community are foreign born, a rate higher than the national average of 13.7%, and 15.5% live in poverty [35, 36].

All participants provided consent prior to participating in the study. All study procedures were approved by the Columbia University Irving Medical Center Institutional Review Board and all research activities were conducted in accordance with the Declaration of Helsinki.

### Exposure variable

The main predictor variable—food insecurity in the prior 30 days—was measured using a 2-item survey, adapted from the validated Hunger Vital Sign™, which aims to identify households at risk for food insecurity [37]. The 2-item survey captured both acute worry of having enough food (“Within the past 1 month have you worried that your food would run out before you got money to buy more?”) and not having resources to acquire food (“Within the past 1 month did the food you bought just not last and you didn’t have money to get more?”). Participants answered either “Often,” “Sometimes,” or “Never” to both questions. Mothers who answered “often” or “sometimes” to either or both items were defined as being at risk for food insecurity. Participants responded to this question upon COMBO baseline enrollment.

### Outcome measures

Main outcomes, which included (i) maternal confidence with infant feeding after hospital discharge and (ii) infant feeding practices, were measured via a self-administered baseline questionnaire at COMBO enrollment at either 1- or 2-months postpartum. Maternal confidence with infant feeding (both formula and breastfeeding) was measured using a 4-item Likert scale adapted from the Breastfeeding Self-Efficacy Scale Short-Form, both validated and reliable in English and Spanish [38, 39]. Responses ranged from “completely confident” to “not at all confident.” Breastfeeding practices were measured from an infant feeding practice question previously developed and tested by CUIMC Newborn Medicine clinicians [39–42], where mothers reported: “all breastmilk,” “mostly breastmilk but some formula,” “equal amounts of breastmilk and formula,” “mostly formula but some breastmilk,” or “all formula” [40, 42]. The 5-level variable was subsequently combined into a 3-level variable of “all breastmilk,” “breastmilk and formula” and “all formula.”

### Covariates

Additional sociodemographic covariates associated with food insecurity risk and breastfeeding practices a priori were abstracted from electronic health records or other self-administered survey questions, including maternal age, infant age, self-reported ethnicity and race, prenatal SARS-CoV-2 infection status, primary language spoken at home, and pandemic wave (early vs. later), insurance status, gestational age, delivery method, infant sex, and education level based on results from other studies [12, 31, 43–45].

### Statistical analysis

All mother-infant dyads with complete food insecurity risk and outcome data were included in the final analytic sample. Bivariate analyses compared covariates and outcomes by food insecurity risk status. Categorical variables were analyzed using Chi-square and Fisher Exact tests and continuous variables were analyzed using Mann-Whitney U tests. Ordinal logistic regression models estimated the association between food insecurity risk and outcome measures significant on bivariate analyses. After assessing for collinearity among variables, models were adjusted for non-collinear a priori covariates and covariates significant at the *p* < 0.25 level during model-building: maternal prenatal SARS-CoV-2 infection status, main language spoken in the home, infant sex, infant age at COMBO enrollment, and maternal age. Secondary analyses included variables for time as early wave (March 2020-December 2021) vs. later wave (January 2022-May 2024) of the pandemic and the interaction between food insecurity risk and time in the adjusted model. January 2022 was selected as this time period corresponds to a distinct shift in the COVID-19 pandemic: Omicron and its sublineages became the predominant circulating variants globally and were associated with record case counts, while vaccine effectiveness against infection had declined relative to earlier phases of the pandemic, reflecting broader changes in transmission dynamics, immunity, and public-health conditions [46, 47]. Given the significance of the time term, the models adjusted by covariates were then stratified by wave of pandemic. Statistical analyses were conducted using RStudio (Version 2024.04.2 + 764, R Core Team, 2025), and statistical significance was at the *P* < 0.05 level.

## Results

Our sample was comprised of *N* = 606 mother-infant dyads who gave birth between March 2020 and May of 2024, of which 40% of dyads had been exposed to prenatal SARS-CoV-2 infection. Sample descriptive statistics are summarized in Table 1. Half (48% [*N* = 289]) of mothers self-identified as Latina, 33% as White (*N* = 200), 10% as Black (*N* = 63), and 4% as Asian (*N* = 27). Mean maternal age was 31.6 ± 5.7 years, mean maternal body mass index (BMI) at delivery was 31.8 ± 6.7 kg/m^2^, and 36% (*N* = 231) mothers delivered via C-section. Mean gestational age for all infants was 38.7 ± 1.9 weeks. More than half of the mothers (52%, *N* = 316) had private insurance.

**Table 1:** Cohort characteristics

Variable	Overall Sample (*N* = 606)	Food Insecure Risk (*N* = 144)	Food Secure (*N* = 446)	*P*-value
**No. (%) or Mean (± Standard Deviation)**				
**Maternal Characteristics**				
** SARS-CoV-2 Exposure**				
Exposed	244 (40%)	60 (42%)	161 (36%)	0.04
Unexposed	289 (48%)	55 (38%)	233 (52%)	
Unknown	73 (12%)	29 (20%)	52 (12%)	
** Language**				
English	464 (77%)	77 (54%)	377 (85%)	< 0.001
Spanish	109 (17%)	64 (45%)	39 (9%)	
Other	22 (4)	1 (1%)	21 (5%)	
** Ethnicity**				
Latino or Hispanic or Spanish Origin	289 (48%)	118 (83%)	171(38%)	< 0.001
Not Latino or Hispanic or Spanish Origin	290 (48%)	24 (17%)	266 (60%)	
** Race (Asian)**				
Asian	27 (4%)	1 (1%)	25 (6%)	< 0.001
Black or African American	63 (10%)	17 (12%)	43 (10%)	
White	200 (33%)	22 (15%)	176 (40%)	
Other	305 (50%)	102 (71%)	193 (43%)	
** Medical Coverage**				
Public	279 (46%)	115 (80%)	150 (34%)	< 0.001
Private	316 (52%)	27 (19%)	287 (64%)	
** Maternal Age at Delivery**	31.6 (± 5.7)	30.0 (± 6.2)	32.2 (± 5.5)	< 0.001
** Maternal BMI at Delivery**	31.8 (± 6.7)	32.1 (± 5.5)	31.8 (± 7.1)	0.24
** Maternal Educational Level**				
7-12th grade	26 (4%)	16 (11%)	10 (2%)	< 0.001
High school degree/GED	42 (7%)	15 (10%)	27 (6%)	
Partial college	52 (9%)	20 (14%)	32 (7%)	
2-year college degree/4-year college degree/Trade school/apprenticeship	154 (25%)	36 (25%)	117 (26%)	
Graduate degree	154 (25%)	14 (10%)	140 (31%)	
Other	4 (1%)	1 (1%)	3 (1%)	
** Infant Characteristics**				
** Baby Sex**				
Male	329 (54%)	87 (60%)	233 (52%)	0.11
Female	277 (46%)	57 (40%)	213 (48%)	
** Mode of Delivery**				
Vaginal	375 (62%)	77 (53%)	286 (64%)	0.03
C-section	231 (36%)	67 (47%)	160 (36%)	
** Number of Children in Household**				
1	163 (27%)	27 (19%)	135 (30%)	< 0.01
2–3	156 (26%)	43 (30%)	113 (25%)	
4+	32 (5%)	12 (8%)	19 (4%)	
** Gestational Age**	38.7 (± 1.9)	38.6 (± 1.7)	38.7 (± 1.8)	0.34
** Birth Weight in Grams**	3255.3 (± 557.6)	3269.6 (± 571.7)	3258.4 (± 543.9)	0.78

Exposed: *in utero* maternal SARS-CoV-2 exposed infants born during the pandemic; Unexposed: Unexposed infants born during the pandemic; Stress Controls: Infants born pre-pandemic in February 2020

*Comparisons performed using Chi Squared Test for binomial variables with expected cell values of five and over, Fisher’s Exact Test for binomial variables with expected cell values under five, and Mann-Whitney U test for continuous variables

*Some data not available for all participants due to nonresponse

Bivariate statistics comparing the relationship between experiencing household food insecurity and demographic characteristics are also summarized in Table 1. Approximately 24% of mothers were at risk for household food insecurity in the last 30 days based on the validated Hunger Vital Sign criteria [37]. There was a significant association between food insecurity risk and prenatal SARS-CoV-2 infection, with mothers who were exposed to prenatal SARS-CoV-2 infection more likely to experience food insecurity in the last month (*p* = 0.04). Greater food insecurity risk was seen among mothers who spoke Spanish (45%) compared to mothers who did not speak Spanish (9%) (*p* < 0.001) and among mothers who self-identified as Latina (83%) compared to mothers who were not Latina (17%) (*p* < 0.001). Mothers with food insecurity risk were more likely to be younger (mean age 30.0 ± 6.2 years vs. 32.2 ± 5.5 years, *p* < 0.001), have public health coverage (80% of mothers with food insecurity risk had public health coverage, while 19% had private coverage, *p* < 0.001), have lower levels of education (*p* < 0.001), and have more children in their households (*p* = 0.01). Mothers who delivered via C-section were also more likely to experience food insecurity risk (*p* = 0.03).

Approximately 54% of the entire sample reported being completely confident in feeding their infant after hospital discharge. Risk for food insecurity was not significantly associated with maternal infant feeding confidence (*p* = 0.06).

In unadjusted models of the entire sample, food insecurity risk was associated with formula feeding (*p* < 0.001). Only 18% of mothers with food insecurity risk exclusively breastfed their child, while 26% exclusively formula-fed. By contrast, 41% of mothers not experiencing food insecurity exclusively breastfed their child, while 13% of them only formula-fed.

In the adjusted model, food insecurity risk was no longer associated with infant feeding practice (adjusted odds ratio [AOR] of exclusively breastfeeding 0.47, 95% confidence interval 0.21, 1.03, *p* = 0.06) (Table 2).

**Table 2:** Infant feeding practice, adjusted OR and CI: Modelling the Adjusted Odds of Formula Feeding vs. Breastfeeding (*n* = 606)

	OR	95% CI	*P*-value
**Variable (reference group)**			
**Food Insecurity Risk Status ** **(Food Secure)**			
Food Insecure Risk	0.47	0.21, 1.03	0.06
**SARS-CoV-2 Infection (Unexposed)**			
Exposed	0.58	0.30, 1.14	0.12
**Maternal Ethnicity (Not of Latino or Hispanic or Spanish Origin)**			
Latino or Hispanic or Spanish Origin	0.21	0.10, 0.43	< 0.001
**Infant’s Adjusted Age**	1.43	1.02, 2.01	0.04
**Maternal BMI**	1.09	1.03, 1.15	< 0.01
**Mode of Delivery ** **(Vaginal)**			
C-section	0.48	0.24, 0.95	0.04

Secondary analyses of the full analytic sample for each outcome included timing of pandemic wave as a covariate (early vs. later pandemic waves: March 2020–December 2021 and January 2022–May 2024). An additional file summarizes the sample descriptive statistics divided by pandemic wave [see Additional file 1]. The interaction between food insecurity risk and pandemic wave was not significantly associated with maternal feeding confidence (*p* = 0.17), nor was wave timing independently associated with this outcome (*p* = 0.16) [see Additional file 2]. However, wave timing was significantly associated with infant feeding method (*p* = 0.02) [see Additional file 3]. Subsequent stratified analysis showed that mothers who gave birth in the early wave of the pandemic were more likely to exclusively breastfeed compared to those who gave birth in the later wave of the pandemic (38% vs. 24%, *p* = 0.004). There was no significant difference in rate of food insecurity risk between the early and later waves (*p* = 0.09). Food insecurity risk was significantly associated with less of a likelihood of breastfeeding in both waves, with AOR = 0.33 (95% CI: 0.15, 0.71, *p* = 0.004) in the early wave and AOR = 0.03 (95% CI: 0.001, 0.32, *p* = 0.01) in the later wave [see Additional file 4].

## Discussion

Almost one-third of the mothers in our sample had risk for food insecurity, a rate higher than the national household food insecurity rate of 11% from 2019 [17]. This suggests that the pandemic exacerbated rates of food insecurity risk in New York City, consistent with reports of increased rates of food insecurity across the nation. In 2022, food insecurity rates were reported to be 16.6% in the Washington Heights and Inwood area [48], still higher than the 11% rate from 2019 [17].

Contrary to our hypothesis, we did not find a significant relationship between food insecurity risk and maternal infant feeding confidence. Maternal feeding confidence has been associated with perception of control during labor [49], past performance (e.g., past breastfeeding experiences with other children), vicarious experiences (e.g., watching other women breastfeed), verbal persuasion (e.g., encouragement from influential others, such as friends, family, and lactation consultants), and physiological responses (e.g., fatigue, stress, and anxiety) [50]. In our sample, mothers experiencing food insecurity were more likely to have more children; this increased parity, and the accompanying accumulated feeding experience may have been a stronger driver of their infant feeding confidence than current experiences of economic hardship with food insecurity.

In the overall sample, food insecurity risk was significantly associated with feeding method on bivariate analysis but not after adjustment for confounders. However, wave-stratified analyses revealed that food insecurity risk was associated with lower adjusted odds of exclusive breastfeeding in both the early and later pandemic waves. This discrepancy suggests that temporal heterogeneity may have obscured meaningful associations in pooled analyses. This suggests that possibility of external social and structural factors not captured by the study that may have dampened the relationship between food insecurity risk status and infant feeding across the entire time period of the overall sample. Potential temporal variations in the external factors, such as changes in social support, NYC- and federal government-specific nutrition assistance programs and other benefit programs, pandemic-related disruptions, and the 2022 formula shortage may have influenced feeding decisions differently across the early and later waves of the pandemic. Pandemic-related milestones—including stay-at-home orders, vaccine rollout, emergency food assistance expansions, and the Child Tax Credit—may have differentially shaped feeding decisions across time. Stratifying by wave may have allowed for detection of patterns influenced by shifting social, economic, and healthcare conditions that were not fully captured by measured covariates.

The 2022 U.S. formula shortage may have impacted breastfeeding rates, with average breastfeeding-initiation rates increasing by 1.96% across 47 states and the District of Columbia [51]. This shortage represents a critical contextual factor that may have influenced feeding practices during the later pandemic wave. Disruptions in formula availability were associated with changes in feeding behaviors, including increased reliance on breastfeeding, mixed feeding, or unsafe practices such as formula dilution [52]. The timing of the shortage coincides with our later wave period and may partially explain the stronger association observed between food insecurity risk and reduced odds of exclusive breastfeeding during this phase.

Our findings align with prior literature demonstrating an association between household food insecurity and lower rates of exclusive breastfeeding. A meta-analysis by Buccini et al. found that food insecurity was associated with reduced exclusive breastfeeding, and the largest included study reported higher exclusive breastfeeding rates among food-secure women [31]. Yet other studies have found no association between food insecurity and exclusive breastfeeding rates [30, 31, 45, 53], highlighting the complexity of feeding decisions under conditions of stress and time constraints related to economic hardship [54, 55], Evidence from natural disasters and conflict settings suggests that stress, exhaustion, limited privacy, and inadequate nutrition may deter breastfeeding—concerns that may similarly apply in the context of a prolonged pandemic [56, 57].

Additional pandemic-related factors may have further influenced feeding practices. Stay-at-home and work-from-home orders may have reduced privacy for breastfeeding, while expanded access to infant formula through programs such as the Supplemental Nutrition Program for Women, Infants, and Children (WIC), which saw a 2.1% increase in use during the first year of the pandemic, and other food emergency programs may have disincentivized women from exclusive breastfeeding [58, 59]. Although breastfeeding is often framed as cost-saving, studies suggest that mothers experiencing food insecurity may perceive breastfeeding as financially burdensome due to the need for increased food intake to support milk production [60]. Heightened financial insecurity during the pandemic may have amplified these concerns [61].

The COVID-19 pandemic’s potential immediate and long-term effects on breastfeeding practices are yet to be fully understood. Consistent with prior research [62, 63], mothers in our sample with confirmed SARS-CoV-2 infection were less likely to breastfeed, despite evidence that COVID-19 is not transmitted through breastmilk [64]. Our findings underscore the importance of integrating food insecurity screening with infant feeding support in both prenatal and postpartum care settings. Given the consistent association between food insecurity and lower odds of exclusive breastfeeding across pandemic waves, healthcare systems should prioritize early identification of food-insecure mothers and provide targeted, culturally responsive lactation support alongside referrals to nutrition assistance programs such as WIC and local food pantries. Feeding support interventions may be most effective when they address not only breastfeeding education and technique, but also mothers’ concerns about nutritional adequacy, milk supply, and the perceived costs of breastfeeding in the context of household food scarcity. The observed differences in associations between food insecurity and infant feeding outcomes across pandemic waves suggest that broader social and policy contexts may have shaped maternal feeding decisions over time. Pandemic-related milestones may have differentially shaped feeding decisions across time. These shifts in structural supports and constraints likely altered access to resources, healthcare, and feeding options available to families during different phases of the pandemic.

Strengths of our study include presenting household food insecurity prevalence and key breastfeeding behavior and infant feeding outcomes among postpartum mothers during the first waves of the COVID-19 pandemic in New York City, one of the earliest pandemic epicenters in the U.S. Limitations include the cross-sectional design, single health system sample, limited power to detect small effect sizes, and reliance on self-reported measures. Wide confidence intervals reported in our tables maybe a function of our sample size, but still yield important clinical information. Additionally, our adapted food insecurity measure captured acute experiences in the prior month rather than longer-term insecurity, and data on participation in nutrition assistance programs, such as the Special Supplemental Nutrition Program for Women, Infants, and Children, were unavailable.

Future research should employ longitudinal designs to assess dynamic changes in food insecurity and feeding practices over time and incorporate qualitative data to better understand maternal feeding expectations and decision-making. Such work will be essential to informing interventions and policies aimed at reducing disparities in breastfeeding and supporting families experiencing food insecurity during public health crises and beyond.

## Supplementary Information

Below is the link to the electronic supplementary material.

Supplementary Material 1

## Data Availability

The datasets used and/or analysed during the current study are available from the corresponding author on reasonable request.
