# Supplementary material for: Effects of food insecurity risk on infant feeding confidence and practices during the COVID-19 pandemic and its waves
Source: Int Breastfeed J. 2026 Apr 11;21:49. doi: 10.1186/s13006-026-00838-5 (PMC13202830; doi:10.1186/s13006-026-00838-5)
Supplement: Supplementary file 1 — Supplementary Material 1 [file 13006_2026_838_MOESM1_ESM.docx]

| **Additional file 1. Demographic Characteristics between COMBO Study Waves** | | | |
| --- | --- | --- | --- |
| **Variable** | **Early Wave**  **(March 2020-December 2021)**  **(N=487)** | **Later Wave**  **(January 2022-May 2024)  (N=110)^¥^** | **P-value** |
|  | **No. (%) or Mean (+/- Standard Deviation)** | |  |
| **Maternal Characteristics** |  |  |  |
| **SARS-CoV-2 Exposure** |  |  |  |
| Exposed | 209 (43%) | 15 (14%) | <0.001 |
| Unexposed | 276 (57%) | 13 (12%) |  |
| Unknown | 0 (0%) | 39 (35%) |  |
| **Language** |  |  |  |
| English | 372 (76%) | 84 (76%) | 0.02 |
| Spanish | 82 (17%) | 26 (24%) |  |
| Other | 22 (5%) | 0 (0%) |  |
| **Ethnicity** |  |  |  |
| Latino or Hispanic or Spanish Origin | 228 (47%) | 70 (64%) | <0.01 |
| Not Latino or Hispanic or Spanish Origin | 248 (51%) | 40 (36%) |  |
| **Race (Asian)** |  |  |  |
| Asian | 21 (4%) | 6 (5%) | 0.20 |
| Black or African American | 46 (9%) | 16 (15%) |  |
| White | 167 (34%) | 29 (26%) |  |
| Other | 242 (50%) | 59 (53%) |  |
| **Medical Coverage** |  |  |  |
| Public | 212 (44%) | 64 (58%) | 0.01 |
| Private | 264 (54%) | 46 (42%) |  |
|  |  |  |  |
| **Maternal Age at Delivery** | 31.6 (± 5.6) | 31.3 (± 6.1) | 0.64 |
| **Maternal BMI at Delivery** | 31.7 (± 6.7) | 32.6 (±6.8) | 0.25 |
|  |  |  |  |
| **Maternal Educational Level** |  |  |  |
| 7-12^th^ grade | 19 (4%) | 7 (6%) | <0.001 |
| High school degree/GED | 24 (5%) | 16 (15%) |  |
| Partial college | 37 (8%) | 14 (13%) |  |
| 2-year college degree/4-year college degree/Trade school/apprenticeship | 134 (28%) | 18 (16%) |  |
| Graduate degree | 128 (26%) | 22 (20%) |  |
| Other | 3 (0.6%) | 1 (0.9%) |  |
| **Number of Children in Household** |  |  |  |
| 1 | 132 (27%) | 31 (28%) | 0.41 |
| 2-3 | 117 (24%) | 39 (35%) |  |
| 4+ | 24 (5%) | 8 (7%) |  |
|  |  |  |  |
| **Food insecurity risk status** |  |  |  |
| Food insecure risk | 112 (23%) | 31 (28%) | 0.09 |
| Food secure | 372 (76%) | 66 (60%) |  |
| **Maternal Infant Feeding Confidence** |  |  |  |
| Not at all confident | 26 (5%) | 6 (5%) | 0.63 |
| A little bit confident | 45 (9%) | 11 (10%) |  |
| Somewhat confident | 148 (30%) | 29 (26%) |  |
| Completely confident | 265 (54%) | 63 (57%) |  |
| Don’t remember | 1 (0.2%) | 1 (0.9%) |  |
|  |  |  |  |
| **Infant Characteristics** |  |  |  |
| **Baby Sex** |  |  |  |
| Male | 267 (55%) | 58 (53%) | 0.77 |
| Female | 220 (45%) | 52 (47%) |  |
| **Mode of Delivery** |  |  |  |
| Vaginal | 302 (62%) | 70 (64%) | 0.83 |
| C-section | 185 (38%) | 40 (36%) |  |
|  |  |  |  |
| **Gestational Age** | 38.7 (±1.8) | 38.7 (± 2.1) | 0.92 |
| **Birth Weight in Grams** | 3260.7 (±553.5) | 3258.6 (± 558.6) | 0.73 |

Exposed: *in utero* maternal SARS-CoV-2 exposed infants born during the pandemic; Unexposed: Unexposed infants born during the pandemic; Stress Controls: Infants born pre-pandemic in February 2020.

*Comparisons performed using Chi Squared Test for binomial variables with expected cell values of five and over, Fisher’s Exact Test for binomial variables with expected cell values under five, and Mann-Whitney U test for continuous variables.

^¥^Some data not available for all participants due to nonresponse and row percentages may not sum to 100%

| **Additional file 2. Relationship between Food insecurity Risk and Maternal Infant Feeding Confidence Inclusive of Pandemic Wave and Food insecurity Risk-Pandemic Wave terms, Adjusted OR and CI: Modelling the Adjusted Odds of Formula Feeding vs. Breastfeeding** | | | | |
| --- | --- | --- | --- | --- |
|  | OR | 95% CI | P-value |  |
| **Variable (reference group)** |  |  |  |  |
| **Food Insecurity Risk Status**  **(Food Secure)** |  |  |  |  |
| Food Insecure | 0.58 | 0.37, 0.88 | 0.01 |  |
| **Wave (Later Wave)** |  |  |  |  |
| Early Wave | 0.57 | 0.26, 1.27 | 0.16 |  |
| **Food Insecurity Risk-Wave Interaction** | 1.93 | 0.75, 4.90 | 0.17 |  |

| **Additional file 3. Relationship between Food insecurity Risk and Infant Feeding Practice Inclusive of Pandemic Wave and Food insecurity Risk-Pandemic Wave terms, Adjusted OR and CI: Modelling the Adjusted Odds of Formula Feeding vs. Breastfeeding** | | | | |
| --- | --- | --- | --- | --- |
|  | OR | 95% CI | P-value |  |
| **Variable (reference group)** |  |  |  |  |
| **Food Insecurity Risk Status**  **(Food Secure)** |  |  |  |  |
| Food Insecure | 0.27 | 0.14, 0.51 | <0.001 |  |
| **Wave (Later Wave)** |  |  |  |  |
| Early Wave | 12.0 | 2.12, 225.88 | 0.02 |  |
| **Food Insecurity Risk-Wave Interaction** | 0.20 | 0.01, 1.35 | 0.16 |  |

| **Additional file 4. Infant Feeding Method, Adjusted OR and CI: Modelling the Adjusted Odds of Formula Feeding vs. Breastfeeding by Wave** | | | | | | |
| --- | --- | --- | --- | --- | --- | --- |
|  | Early Wave | | | Later Wave | | |
|  | OR | 95% CI | P-value | OR | 95% CI | P-value |
| **Reference Group** |  |  |  |  |  |  |
| **Food Insecurity Risk Status (Food Secure)** |  |  |  |  |  |  |
| Food Insecure Risk | 0.33 | 0.15, 0.71 | 0.004 | 0.03 | 0.001, 0.32 | 0.01 |
| **Infant’s Adjusted Age** | 1.37 | 0.98, 1.92 | 0.06 | 0.18 | 0.03, 0.78 | 0.04 |
| **Maternal BMI** | 1.11 | 1.05, 1.17 | <0.001 | 1.18 | 1.05, 1.38 | <0.001 |
| **Maternal Age** | 0.89 | 0.84, 0.95 | <0.001 | 0.88 | 0.76, 0.99 | 0.05 |
| **Mode of Delivery (Vaginal)** |  |  |  |  |  |  |
| C-section | 0.39 | 0.19, 0.78 | <0.01 | 0.26 | 0.05, 1.27 | 0.11 |
